# SLIPS-TENG: robust triboelectric nanogenerator with optical and charge transparency using a slippery interface

**DOI:** 10.1093/nsr/nwz025

**Published:** 2019-03-01

**Authors:** Wanghuai Xu, Xiaofeng Zhou, Chonglei Hao, Huanxi Zheng, Yuan Liu, Xiantong Yan, Zhengbao Yang, Michael Leung, Xiao Cheng Zeng, Ronald X Xu, Zuankai Wang

**Affiliations:** 1 Department of Mechanical Engineering, City University of Hong Kong, Hong Kong, China; 2 Department of Precision Machinery and Precision Instrumentation, University of Science and Technology of China, Hefei 230026, China; 3 Shanghai Key Laboratory of Multidimensional Information Processing, Department of Electronic Engineering, East China Normal University, Shanghai 200241, China; 4 Department of Chemistry, University of Nebraska-Lincoln, Lincoln, NE 68588, USA; 5 School of Energy and Environment, City University of Hong Kong, Hong Kong, China; 6 Department of Materials Science and Engineering, City University of Hong Kong, Hong Kong, China

**Keywords:** slippery surface, triboelectricity, power generation, water droplet, interfaces

## Abstract

Energy harvesting devices that prosper in harsh environments are highly demanded in a wide range of applications ranging from wearable and biomedical devices to self-powered and intelligent systems. Particularly, over the past several years, the innovation of triboelectric nanogenerators (TENGs) that efficiently convert ambient kinetic energy of water droplets or wave power to electricity has received growing attention. One of the main bottlenecks for the practical implications of such devices originates from the fast degradation of the physiochemical properties of interfacial materials under harsh environments. To overcome these challenges, here we report the design of a novel slippery lubricant-impregnated porous surface (SLIPS) based TENG, referred to as SLIPS-TENG, which exhibits many distinctive advantages over conventional design including optical transparency, configurability, self-cleaning, flexibility, and power generation stability, in a wide range of working environments. Unexpectedly, the slippery and configurable lubricant layer not only serves as a unique substrate for liquid/droplet transport and optical transmission, but also for efficient charge transfer. Moreover, we show that there exists a critical thickness in the liquid layer, below which the triboelectric effect is almost identical to that without the presence of such a liquid film. Such an intriguing charge transparency behavior is reminiscent of the wetting transparency and van der Waals potential transparency of graphene previously reported, though the fundamental mechanism remains to be elucidated. We envision that the marriage of these two seemingly totally different arenas (SLIPS and TENG) provides a paradigm shift in the design of robust and versatile energy devices that can be used as a clean and longer-lifetime alternative in various working environments.

## INTRODUCTION

The continuous prosperity and economic growth in humankind demands new strategies to combat the grand energy challenge [1–6]. One promising solution is the scavenging of abundant, clean and renewable water-related energy [7–11]. At the large scale, the construction of hydroelectric plants at specific areas such as river dams has achieved significant success in transforming water's potential energy into electricity [12]. Equally important as this bulk water power but receiving less attention is the limitless energy stored in ubiquitous water droplets, which are manifested in the form of raindrops, waterfalls, dewdrops, etc. Taking raindrops as an example, the annual water precipitation worldwide is ∼5.05 × 10^5^ km^3^, which could result in a power of ∼0.5 TW if the total kinetic energy of these droplets can be fully translated into electricity [13,14]. However, to date, such a huge amount of energy has not been successfully harvested because of the lack of disruptive, efficient and scalable technologies.

Triboelectricity is one of the oldest and most fascinating findings, dating back to experiments by the ancient Greek philosopher Thales of Miletus, who discovered that rubbing amber against wool gave rise to electrostatic charging. Currently, triboelectricity has been extended to different technological applications such as electrostatic separation, photocopying, and laser printing [15–18]. In 2012, Wang *et al.* reported the development of triboelectric nanogenerators (TENGs) and subsequently spurred the pursuit of sustainable blue energy [19–21]. Despite the diversity in their design or applications, the basic working mechanism of TENGs relies on the combined effects of triboelectricity [22] and electrostatic induction, which take place either at the solid/solid or solid/liquid interfaces.

From the practical application perspective, the development of ideal TENGs that function well in a wide range of working conditions remains elusive. Currently for the solid/liquid interface-based TENG, the solid phase is designed to be hydrophobic or superhydrophobic (SHS) so that water droplets can be repelled in a timely manner to refresh contact sites for continuous power generation [23]. However, the adoption of SHS-based TENGs, referred to as SHS-TENGs, leads to the rapid bouncing of impinging water droplets [24–26], significantly reducing the effective solid/liquid contact area and thereby compromising the energy conversion efficiency. Moreover, to dramatically improve the energy conversion efficiency, a higher droplet impact velocity is preferred [27]. However, the adoption of a higher velocity naturally induces high dynamic pressure, leading to the collapse of the preferred long-term liquid repellency as well as unwanted instability in power generation. Similarly, when exposed to dynamic working conditions involving mechanical stretching, bending, and abrasion, the rough structures essential to the SHS state of TENGs can be easily broken [28]. Subsequently the actual output performance as well as the optical transparency is degraded [29].

More challenges emerge when these SHS-TENGs are exposed to extreme environments such as high humidity, low temperature and submerged conditions. In the first two conditions, the nucleation of nanoscale water droplets or ice/frost dramatically suppresses the mobility of impinging droplets, eventually resulting in the freezing of the entire surface and screening the effective charge generation and transport [30]. Similarly, when used for wave energy harvesting in submerged conditions, the longevity of SHS-TENGs is susceptible as a result of unwanted wetting transition and biofilm formation [31]. In all these cases, the collapse of SHS and fouling of SHS-TENGs surface significantly compromises the energy harvest efficiency [32].

Herein we report a new slippery lubricant-impregnated porous surface (SLIPS) [33–40] based TENG that exhibits many promising advantages over conventional design including optical transparency, configurability, self-cleaning, flexibility and power generation stability, even under harsh environments. We demonstrate that the SLIPS not only serves as a unique substrate for liquid/droplet transport and optical transmission, but also enables a charge transparency-like triboelectricity through a unique liquid/SLIPS interface. As a result of such intriguing property, we show that under a low operating temperature environment, the output power of the SLIPS-TENG is at least an order of magnitude higher than that of the conventional SHS-TENG.

## RESULTS AND DISCUSSION

**Figure 1.**
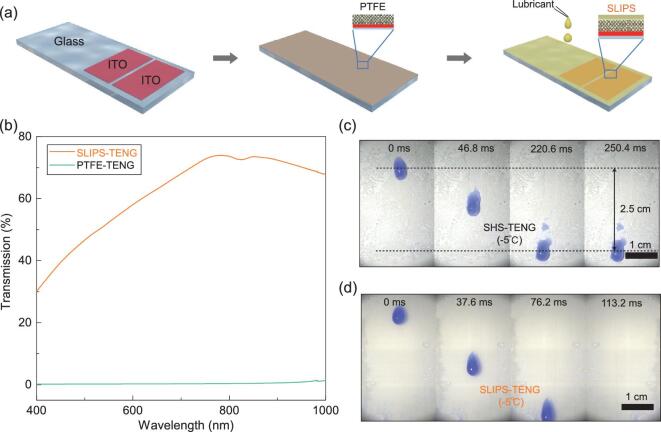
SLIPS-TENG fabrication and optical and wetting property characterization. (a) Schematic drawing of the SLIPS-TENG fabrication process. (b) Comparison of optical transmittance between SLIPS (SLIPS-TENG) and PTFE (SHS-TENG). (c, d) The enhanced anti-icing capability is also evidenced under droplet impact conditions at freezing temperature. A droplet on SHS (c) is easily pinned because of the formation of an ice layer and then becomes frozen at the pinning position on SLIPS. (d) The impinging droplet slides off in a timely manner.

To construct the SLIPS-TENG, we first prepare patterned indium tin oxide (ITO) electrodes on transparent glass slide, followed by the gentle deposition of the SLIPS layer (see Fig. 1a and the detailed description in the ‘Methods’ section). For the SLIPS fabrication, we choose a porous PTFE membrane with an average pore size of 200 nm and thickness of 20 μm as the matrix because PTFE is the most electronegative material in the triboelectric series. A perfluorinated liquid (DuPont Krytox GPL103, surface tension *γ* ≈ 16–20 mN/m) is chosen as the lubricant owing to its many advantages such as high wetting affinity with PTFE membrane, immiscibility with most liquids, high temperature stability, low vapor pressure, chemical inertness, and compatibility with various materials (Supplementary Fig. 1). Upon placing the lubricant on the PTFE surface, it wets spontaneously into the membrane via capillary wicking owing to the preferential affinity with the PTFE membrane. The thickness of the over-coated lubricant layer (*h_0_*) on the PTFE membrane is maintained at ∼2 μm. As shown in Supplementary Figs 2 and 3, the as-fabricated SLIPS-TENG demonstrates exceptional self-cleaning and anti-wetting properties to various liquids.

As expected, the SLIPS-TENG also exhibits enhanced optical transparency in the visible light wavelength range as a result of reduced light scattering through the SLIPS-TENG enabled by the replacement of the unwanted solid/air interface with a liquid/air interface (Fig. 1b). In addition to amplifying the optical transparency, the infusion of the lubricant layer also results in preferential interfacial stability, especially in environments involving freezing temperature and high humidity. At a temperature of −5°C and ambient relative humidity of 43%, ice is supposed to nucleate and propagate on SHS surfaces. Notably, we find that even under the freezing temperature, the SLIPS surface maintains a superior stability. This is because the replacement of air pockets with the liquid lubricant in SLIPS eliminates contact line pinning and random surface defects, which tend to trigger the formation of ice crystals and the rapid propagation of ice crystal waves over the entire surface. In contrast, on the control experiment using SHS-TENG, the superhydrophobic state of the surface is easily broken down owing to the formation of ice/frost at the random defects, and then the entire surface becomes frozen within 5 min. Such an enhanced anti-icing capability enabled by the use of SLIPS is also evidenced under droplet impact condition below freezing temperature (−5°C). As shown in Fig. 1c, when a 25 μL water droplet impinges on supercooled SHS substrate with a tilt angle of 45°, it finally gets pinned after a sliding motion along the SHS with a distance of 2.5 cm. However, on SLIPS-TENG, the droplet completely slides away without any contact line pinning (Fig. 1d). Taken together, the construction of TENG with the slippery lubricant layer endows enhanced wetting and optical properties in a wide range of conditions.

**Figure 2.**
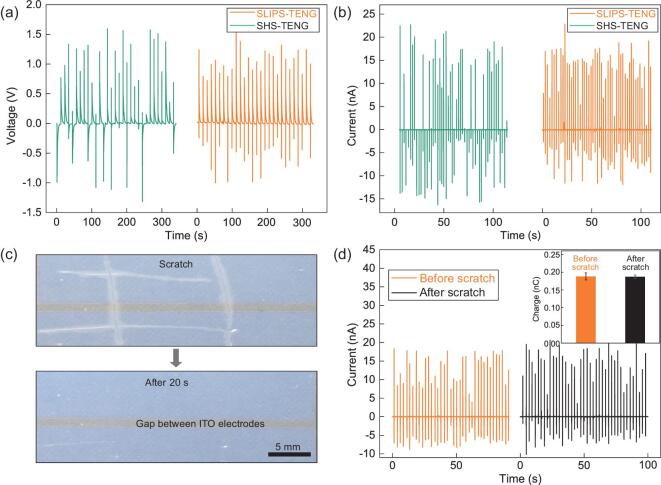
Electricity generation and reconfigurable behavior of SLIPS-TENG. (a, b) Comparison of open-circuit voltage and short-circuit current between SLIPS-TENG with SHS-TENG at room temperature of 25°C under a fixed droplet size of 100 μL. (c) The PTFE matrix is first damaged with several scratches (top) and within 20 s, the smooth interface and optical property of SLIPS-TENG can be restored (bottom). (d) Comparison of the measured short-circuit current for SLIPS-TENG before and after the creation of scratches. The inset shows the measured charge from an impacting water droplet after contact electrification with the SLIPS-TENG before and after scratching.

Unexpectedly, we find that the design of the slippery liquid layer also gives rise to superior electricity generation capability. Figure 2a and b shows the comparison of measured electric outputs from SLIPS-TENG and SHS-TENG at room temperature (25°C), respectively. On both devices, the releasing height and frequency of incoming droplet chains is 10 cm and 0.1 Hz (Supplementary Fig. 4), respectively. The generated open-circuit voltage and short-circuit current are measured to be about 1.2 V and 20 nA, respectively, indicating that the replacement of air pockets with appropriate lubricant liquid will not completely screen the electricity generation. Notably, within the same testing period, the amount of peaks in the voltage and current output from SLIPS-TENG is much larger than that from SHS-TENG, indicating the enhanced stability in the electrical output as a result of the decoration of unique water/SLIPS interface. The enhanced stability can also be explained by careful inspection of droplet dynamics on different substrates. Statically, the base area of a droplet in contact with SLIPS is about 7.7 times larger than that on SHS (Supplementary Fig. 5). Dynamically, a droplet always stays in intimate contact with SLIPS-TENG during the entire process, as shown in Supplementary Fig. 6a and c. In contrast, a droplet hitting SHS-TENG easily bounces off without the preferential contact with both electrodes (Supplementary Fig. 6b and d, Supplementary Movie 1). Moreover, we also show that the electricity generation in SLIPS-TENG is not sensitive to physical damage of the underlying PTFE matrix. To demonstrate this, we first create several damage sites using the knife-scratch method, which can be clearly seen from the optical image shown in Fig. 2c. Interestingly, within 20 s the SLIPS-TENG interface restores its original optical property owing to the infusion of lubricant into the damage sites. Despite this, we find that the creation of scratches has a negligible effect on the energy generation. As shown in Fig. 2d, both the short-circuit currents measured from SLIPS-TENG with scratches are comparable to those from the original SLIPS-TENG, indicating a remarkable robustness owing to the softness and configurability of the lubricant.

**Figure 3.**
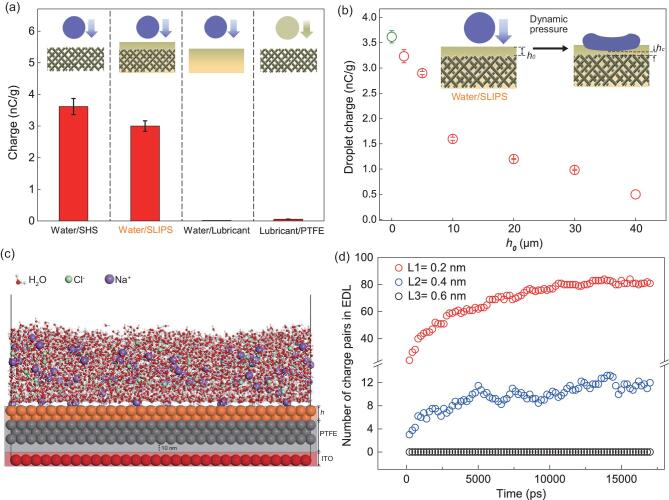
Charge transparency behavior of SLIPS-TENG. (a) Comparison of the charge obtained from a droplet hitting on various interfaces including the water/SHS, water/SLIPS, water/lubricant and lubricant/PTFE interfaces, respectively. The charges generated on SHS and SLIPS are substantially larger than those of the water/lubricant and lubricant/PTFE interfaces. (b) The variation of the measured charge in a droplet after contact electrification with the SLIPS-TENG as a function of the lubricant layer thickness *h*_0_. (c) Molecular dynamics simulation to mimic the SLIPS-TENG with a lubricant layer of thickness *h_0_*. Here, Na^+^ and Cl^−^ ions are dissolved in the top water layer to simulate the charge transfer after water droplets hit on the TENG. The water layer is in contact with a coating layer (orange spheres) with thickness *L*. (d) Variation of the amount of EDL pairs in EDL formed at the water/SLIPS interface with time progression with different thicknesses (L1, L2, and L3 are 0.2 nm, 0.4 nm, and 0.6 nm, respectively) of the coating layer.

To further probe the basic mechanisms responsible for the intriguing electricity generation at the water/SLIPS interface, we continued to investigate the roles of various interfaces during the triboelectricity process. To rule out the possibility that the electricity generation results from the droplet itself, we first measured the charge of a falling droplet with and without contact with TENG using a Faraday cup (Supplementary Fig. 7), respectively. As shown in Fig. 3a, under a fixed release height of 10 cm, the measured charge from the droplet falling directly into the Faraday cup is 0.055 nC/g, which is two orders of magnitude smaller than that obtained from the droplet impinging on SLIPS-TENG (3 nC/g). The striking contrast in the droplet charges in two different cases convincingly suggests that the charge generation and transfer should mainly originate from the water/SLIPS interface and the effects of both water/needle and water/air interfaces are negligible. Moreover, as shown in Fig. 3a, the generated charges from an impinging water droplet on a thin lubricant film is measured to be ∼0.018 nC/g, which is comparable to that from a lubricant droplet hitting a PTFE matrix (∼0.055 nC/g). Notably, in both cases, the measured charges are insignificant compared to that on SLIPS-TENG, indicating that the triboelectricity manifested on the water/lubricant interface or lubricant/PTFE interface is negligible [41]. Taken together, all these experiments clearly suggest that the charge generation obtained from SLIPS-TENG still originates from the triboelectricity between water and the PTFE matrix. It is also important to note that an optimal charge transfer between water and SLIPS also demands a proper choice of the type and viscosity of lubricant (Supplementary Fig. 8), though the specific mechanisms remain to be uncovered in future study.

We further validate that during the entire process, the thin film can always keep an integral configuration without rupture. Previously it has been shown that, owing to the high wetting affinity between the lubricant and PTFE matrix, SLIPS can maintain a steady state even under a dynamic water pressure higher than 5 kPa [33,42]. Despite the difficulty in measuring the thickness of the lubricant layer due to its soft and self-configurable nature, the time evolution of lubricant layer thickness $h( t )$ can be quantitatively estimated based on a thin film lubrication approximation analysis, which can be expressed as

(1) \begin{equation*}h( t ) = \frac{1}{{\sqrt {\frac{{\pi \rho v_i^2}}{{{\mu _L}{r^2}}}t + \frac{1}{{h_0^2}}} }},\end{equation*}

where ${\mu _L}$ is the dynamic viscosity of the lubricant, ${h_0}$ is the initial thickness of the lubricant film, and $r$, $\rho $ and ${v_i}$ represent the radius, density and velocity of impacting water droplet respectively. For a typical dynamic pressure of 1000 Pa (scaled as $P\sim \rho v_i^2/2$) and timescale of 1.7 ms $(t\sim r/{v_i})$, the minimal thickness *h_0_* in the deformed lubricant layer is still much larger than the van der Waals length scale (∼100 nm, Supplementary Fig. 9), below which the attractive force between the water and PTFE becomes vital to break down the thin lubricant film [43]. In coupled with the above charge measurement, our results demonstrate that the unique liquid layer exhibits a charge transparency-like behavior, which is reminiscent of the wetting transparency [44,45] and van der Waals potential transparency of graphene previously reported, although the specific mechanisms remain to be explored [46].

We also demonstrate that the manifestation of the charge transparent-like behavior in SLIPS-TENG demands an exquisite control of the thickness *h_0_* of the lubricant. As shown in Fig. 3b, the measured net charge of 60 μL droplet on the SLIPS-TENG sample displays a gradual decay with the increase of lubricant layer thickness *h_0_*. Thus, given that the thickness of the lubricant liquid layer is smaller than a threshold value *h_c_*, the presence of the slippery liquid layer exerts an insignificant effect on the triboelectricity between a water droplet and SLIPS. To further understand the thickness-dependent electricity generation between water and SLIPS, we further conducted a molecular dynamics (MD) simulation by using a scaled-down model system, in which the negative charges are fixed on the topmost surface of PTFE, as depicted in Fig. 3c and Supplementary Fig. 10. Due to electrostatic interactions with the negative charges on the PTFE surface, an electrical double layer (EDL) is formed as cations in the water droplet are preferentially attracted on the surface of the lubricant coating layer (Supplementary Movie 2). As shown in Fig. 3d, the total number of accumulated charge pairs in the formed EDL increase with decreasing the thickness of the thin lubricant coating layer, suggesting that the electricity generation capacity of SLIPS-TENG is indeed intricately regulated by the thickness of the lubricant layer.

After elucidating how the triboelectricity is regulated by the water/SLIPS interface, we continue to investigate the stability of SLIPS in its long-term operation. Supplementary Fig. 11 plots the variation of electric current as well as the lubricant loss relative to the number of droplets impinging. Here the lubricant loss is defined as the reduced weight of the lubricant relative to its original value. The experiment is conducted in ambient conditions with a temperature of 24°C ± 1.6°C and relative humidity of 42.7% ± 3.4%. As shown in this figure, there is only 0.4% loss after 10 000 consecutive droplet impingements and there is only a slight increase in the average electric current output during the entire testing, suggesting the remarkable stability of the lubricant layer.

**Figure 4.**
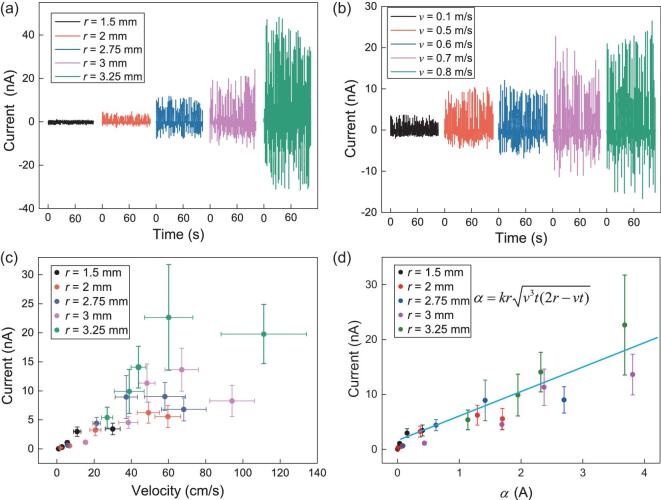
Effects of droplet size and velocity on the electricity generation of SLIPS-TENG. (a, b) Variation of current peak under different *r* (a) and sliding velocity of droplets *v* (b). (c) The distribution of electrical current under different *r* and *v.* (d) Linear fitting between current and the droplet size, sliding velocity. The random data shown in (c) are collapsed into a linear curve, showing the validity of our scaling analysis.

We also construct a simple analytical model to quantify how the electricity is influenced by the water droplet itself. Figure 4a and b shows the variation of current peak under different radii (*r*) and sliding velocities of droplets (*v*). Based on these experimental results, we also developed a simple scaling model that can capture the physical essence (see the detailed description in the ‘Methods’ section). Here we assume that: (1) the droplet/SLIPS-TENG contact base is a circle with an area *S*; (2) the charge is induced at the droplet/SLIPS-TENG interface, and the amount of charge *Q* is proportional to the droplet volume Ω, i.e. *Q* = *k*Ω, where *k* is a pre-factor. Thus, the output current in the circuit can be expressed as: *I*(*t*) = *dq*(*t*)/*dt* = (*Q*/*S*)⋅*dS*_c_(*t*)/*dt, w*here *q*(*t*) is a time-dependent induced charge on the ITO electrode, and *S_c_*(*t*) represents the time-dependent solid/liquid overlapping area, respectively. Finally, the current is written as

(2) \begin{equation*}I(t)\sim kr\sqrt {{v^3}t(2r - vt)} .\end{equation*}

After a scaling analysis using the above model, all the current data points shown in Fig. 4c collapse into a linear curve, suggesting the validity of our model (Fig. 4d).

**Figure 5.**
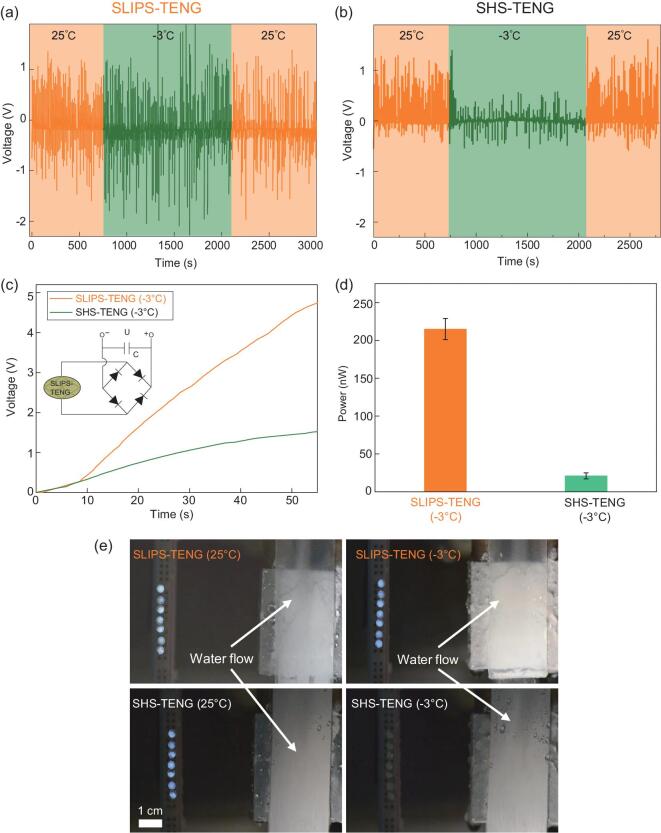
Enhanced stability in the electricity generation of SLIPS-TENG at low temperature (−3°C). (a) The time-dependent variation of the open-circuit voltage of SLIPS-TENG at 25°C and −3°C, respectively. SLIPS-TENG shows an enhanced stability of electrical output due to the super-slippery interface. In contrast, upon the lowering the temperature below the freezing point, small condensate droplets are formed and get pinned on SHS, preventing the effective charge separation in the triboelectricity process. Here the droplet volume is 100 μL and continuous droplet arrays impact on the device. (b) The open-circuit voltage of SHS-TENG at 25°C and −3°C, respectively. After the substrate temperature is switched to −3°C, the output voltage is reduced from 1 V to 0.3 V. SLIPS-TENG can maintain a relatively high output voltage of 1.2 V, whereas the performance of SHS-TENG is severely degraded at low temperature. (c) Plots of time-dependent charged voltages across a 1 μF capacitor in SLIPS-TENG and SHS-TENG. Within a period of 55 s, the capacitor in the SLIPS-TENG can charge to 5 V, which is much higher than of the SHS-TENG. (d) The measured output power of SLIPS-TENG is 200 nW, which is one order of magnitude larger than that of SHS-TENG. (e) Photographs showing the lighting-up of LED bulb arrays by the continuous flow of water droplets on SLIPS-TENG and SHS-TENG at 25°C and −3°C, respectively. The SLIPS-TENG device can light up bulb arrays at both 25°C and −3°C; however, SHS-TENG fails to function at −3°C.

To demonstrate how the stability and robustness of SLIPS-TENG is translated into superior electricity power harvesting even at low temperature, we further characterize the open-circuit voltage output from free-falling droplet arrays. In our experiment, a syringe pump is used to precisely generate droplets with volume 100 μL and the release frequency of droplets was about 0.1 Hz. At room temperature of 25°C, the open-circuit output voltage measured from both SLIPS-TENG and SHS-TENG are about 1.2 V (Fig. 5a and b). As shown in Fig. 5a, when the temperature is reduced below the freezing point of water, SLIPS-TENG can still maintain a stable output voltage of 1.2 V, which is comparable to that obtained at room temperature. Such an enhanced stability of SLIPS-TENG in freezing temperature conditions is ascribed to the decoration of the smooth and super-slippery liquid/SLIPS interface that delays ice/frost formation and enhances droplet mobility. Indeed, after 1 h only 20% of the whole surface area is covered by ice, and there is no ice accumulation on the pathway of sliding droplets at the central area of our sample. In contrast, the surface ice coverage ratio on the SHS-TENG is increased to about 50% within 15 min (Supplementary Fig. 12). Even before the ice formation, small condensate droplets are randomly formed and get pinned on SHS, which prevent the effective charge separation in the triboelectricity process between the droplet and substrate, as evidenced by the lower output even at the beginning of the temperature drop (Fig. 5b). We also measure the output characteristics under various load resistances to determine the maximum power output. The open-circuit voltage increases from 0 V to 0.7 V and the short-circuit current decreases from 23 nA to 3.7 nA with the load resistance increasing from 1 KΩ to 1 GΩ. The maximum power output is 2.5 nW when the load resistance is set at 100 MΩ (Supplementary Fig. 13). To further demonstrate the advantage of our SLIPS-TENG in practical applications operating in a freezing environment, we further compared the charging capability in a typical full-wave rectifier under continuous water flow, which can generate a current output of 400 nA (Supplementary Fig. 14). Within a period of 55 s at −3°C, the SLIPS-TENG can charge a 1 μF capacitor to 5 V, which is much larger than that in the case of SHS-TENG (Fig. 5c). Moreover, the output power from SLIPS-TENG is 200 nW, which is also one order of magnitude larger than that from SHS-TENG, as shown in Fig. 5d. Such a difference is also evidenced by the lighting experiment, as shown in Fig. 5e. The SLIPS-TENG device can light up bulb arrays at both 25°C and −3°C, whereas there is no obvious lighting at −3°C in the case of SHS-TENG (Supplementary Movies 3 and 4).

The enhanced stability and robustness inherent in SLIPS-TENG are also general, which can be extended to various wearable and flexible devices to impart more versatile functionalities. As a proof of concept demonstration, we fabricate a SLIPS-TENG device on a soft and flexible substrate. Briefly, we first fabricate the top SLIPS layer as well as the underlying soft substrate consisting of patterned thin copper tape and a PDMS layer using the methods discussed above. Then, a thin layer of sticky PDMS oligomer is used to stably bind these two layers. Under a bending state, the device still lights up the LED bulb arrays with a measured output power ∼200 nW, which is comparable to that on the flat SLIPS-TENG (Supplementary Movie 5). The high optical transmittance enabled by the use of a slippery surface also makes it possible to integrate SLIPS-TENG with other optoelectronic devices such as solar cells for collecting both the droplet energy and solar energy simultaneously. Probably more importantly, the marriage of SLIPS and TENG opens up a new avenue for the rational design of novel energy devices that are capable of scavenging abundant wave energy with long-term stability and durability in wet conditions.

## METHODS

### Materials

Acetone (RCI Labscan, 99,5%), ethanol (Sigma Aldrich, 97%), nitric acid (Sigma Aldrich, 70%), hydrochloric acid (Sigma Aldrich, 37%), deionized water, and DuPont Krytox GPL 103 were used without further purification. The dimensions of the purchased indium tin oxide (ITO) glass slides are 2.5 cm × 7.5 cm × 2.5 mm. The thickness and average pore size of the porous PTFE membrane (Sterlitech Corporation, PTU023001) are 25–50 μm and 200 nm, respectively.

### Fabrication of SLIPS-TENG

To fabricate the SLIPS-TENG, a piece of ITO glass slide was first ultrasonically cleaned in acetone and ethanol for 10 min, respectively. Then two 2 cm long Kapton tapes were parallelly attached onto the ITO glass slide by maintaining a gap of 1 mm. After completely etching away the exposed ITO on the as-prepared glass slide with the etching liquid (HNO_3_: HCL: H_2_O = 2: 25: 25) and removing the tape, we gently covered a PTFE membrane onto the etched glass slide. To ensure a good contact between the PTFE membrane and glass substrate, the membrane was first wetted by ethanol with the help of the capillary wicking effect. After the evaporation of the ethanol, several droplets of low-surface-tension DuPont Krytox GPL 103 (γ = 16–20 mN/m) were dripped onto the membrane to wick into the pores of the PTFE. After the addition of lubricant oil to create SLIPS-TENG, all the samples were placed horizontally on a flat table for about 12 hours to allow spontaneous impregnation of lubricant prior to any experimental characterization or measurement. We controlled the thickness of lubricant layer *h* by the control of the volume of lubricant according to the equation $h = V/A$, where *A* is the area of the PTFE membrane.

### Characterization and electrical measurement

The optical transmittance was measured by using a Perkin Elmer Lambda 35 UV-VIS spectrometer. The droplet dynamic behavior of the water droplet was recorded by a Photron FASTCAM SA4 at a rate of 3000 frames per second. A precise source meter, Keithley 2400, was used to measure the electrical outputs of SLIPS-TENG. If not specified, the substrate tilt angle and the release height of the water droplet were fixed at 45° and 10 cm, respectively. The charges of the water droplet and lubricant droplet were measured using a Faraday cup connected with a nanocoulomb meter (MONROE Model 284). To quantify the electricity generation under different initial kinetic energies, the droplet radius was controlled by a plastic tube with different outlet sizes, and the sliding velocity of the droplet was varied by adjusting the substrate tilt angle from 10° to 60°. In particular, for testing the behavior of TENGs at low temperature, any sample (SLIPS-TENG or SHS-TENG) was stuck to a thermoelectric cooling stage, which was used to precisely control the temperature with a cooling rate of around 8°C/min. It took ∼40 s for both SHS-TENG and SLIPS-TENG to reach a steady surface temperature state and the steady surface temperature for SHS-TENG and SLIPS-TENG was very close to that of the cooling stage (Supplementary Fig. 15). All the experiments were carried out under an ambient relative humidity of 43%.

### Molecular dynamics simulations

As the water droplet slides over an electrode, the EDL formed at the water/SLIPS interface creates a potential difference and induces charge transfer across the two electrodes in the circuit (Supplementary Fig. 6). Therefore, how effectively the EDL can be formed at the interface determines the efficiency of electricity generation. To simulate the charge mobility during the process of water droplets impacting with the SLIPS, an equal amount of sodium ions (Na^+^) and chlorine ions (Cl^−^) are introduced into water in the MD simulations. The MD system includes 5000 water molecules with 100 Na^+^ and 100 Cl^−^ being dissolved in the water layer. To mimic the experimental setup, a rigid and smooth hydrophobic atomic layer was used to simulate the coating layer, which is located on top of the PTFE layer and ITO electrode. 100 negative charges and 100 positive charges were fixed, respectively, on the topmost layer of PTFE and the bottom layer of electrode on ITO, and each site was charged ±18e. Water molecules with dissolved Na^+^ and Cl^−^ ions were initially equilibrated for 3 ns of MD simulations at 300 K without charge on the substrates. Next, 17 ns MD simulations in the *NVT* ensemble at 300 K with equivalent positive and negative charges on the substrate were carried out. The box size of the model is 8.65 nm × 8.65 nm × 31.4 nm. Periodic boundary conditions were applied in the *x* and *y* directions. The TIP4P/ICE water model was employed in the MD simulation [47], and the parameters for Na^+^ and Cl^−^ were taken from previous studies [48] (σ_Na_ = 2.876 Å, ϵ_Na_ = 0.5216 kJ/mol, σ_Cl_ = 3.785 Å, ϵ_Cl_ = 0.5216 kJ/mol). The cross Lennard-Jones (LJ) interaction parameters between water and sodium and chlorine ions were given by the Lorentz–Berthelot rule. The interactions between substrate atoms and NaCl water solution were described by a 12–6 LJ potential (σ_Na-sub_ = 3.021 Å, ϵ_Na-sub_ = 0.4785 kJ/mol, σ_Cl-sub_ = 3.476 Å, ϵ_Cl-sub_ = 0.4785 kJ/mol, σ_O-sub_ = 3.458 Å, ϵ_O-sub_ = 0.6223 kJ/mol). The fast smooth particle-mesh Ewald method was used for electrostatic interactions with a real-space cut-off of 10 Å. The van der Waals interactions were truncated at 10 Å. A velocity Verlet algorithm for integrating Newton's equations of motion with a time step of 1 fs was employed in the MD simulations. The constant temperature was controlled using the Nosé–Hoover scheme. All the MD simulations were performed by using the Gromacs 4.5.5 software.

### Theoretical analysis of SLIPS-TENG output current

We also conducted a simple theoretical model to predict the dependence of electrical current on the droplet size and sliding velocity. When a water droplet hits the SLIPS, an electric double layer (EDL) is created at the water/SLIPS interface. Prior to the sliding of the droplet over any electrode, the potential between the upper electrode and the lower electrode is equivalent. Once the water droplet is in contact with the upper electrode, the potential in this upper electrode increases due to the formation of EDL, and electrons are driven under electrostatic induction to the upper electrode from the lower electrode to reach equilibrium. Then, the potential of the upper electrode decreases as the droplet slides from the upper electrode toward the lower electrode due to the shift in the EDL region; electrons are transferred from the upper electrode to the lower electrode to recover equilibrium. When the droplet moves away the lower electrode, electrons are transferred from the lower electrode to the upper electrode to reach equilibrium (Supplementary Figs 6 and 16). A continuous output is achieved with the sequential droplet impinging onto the SLIPS.

Simplistically, we assume that the generated charges in the droplet are uniformly distributed at the droplet/SLIPS-TENG interface and the contact line between SLIPS-TENG and droplet is a circle. At the first stage, the overlapping area between the droplet base and the underlying electrode can be expressed as:

(3) \begin{eqnarray*} {S_{CH}}(t) &=& \frac{{\pi {R^2}}}{2} - (R - vt)\sqrt {{R^2} - {{(R - vt)}^2}}\nonumber\\ && - {R^2}\arcsin \left( {\frac{{R - vt}}{R}} \right),\end{eqnarray*}

where $v$ is the sliding velocity of the droplet and *R* is the radius of the contact line. Thus, the electrical current from the electrode can be expressed as:

(4) \begin{eqnarray*} {I_1}(t) &=& \frac{{dq(t)}}{{dt}} = \frac{Q}{S}\frac{{d{S_{CH}}(t)}}{{dt}}\nonumber\\ && = \frac{Q}{S}\sqrt {{v^3}t(2R - vt)}\nonumber\\ &&\sim {{ k}}R\sqrt {{v^3}t(2R - vt)}. \end{eqnarray*}

Similarly, at the second stage, the electrical current from the electrode can be expressed as:

(5) \begin{eqnarray*} {I_2}(t) &=& \frac{Q}{S}\frac{{d({S_{CL}}(t) - {S_{CH}}(t))}}{{dt}}\nonumber\\ = - 2\frac{Q}{S}\sqrt {{v^3}t(2R - vt)}\nonumber\\ \sim kR\sqrt {{v^3}t(2R - vt)} .\end{eqnarray*}

At the third stage, the electrical current from the electrode can be expressed as:

(6) \begin{eqnarray*}{I_3}(t)&=&\frac{Q}{S}\frac{{d{S_{CL}}(t)}}{{dt}} = \frac{Q}{S}\sqrt {{v^3}t(2R - vt)} \nonumber\\ &&\sim kR\sqrt {{v^3}t(2R - vt)} .\end{eqnarray*}

 

Thus, because of *r ∼ R*, combining these results yields the scaling law for the output current:

(7) \begin{equation*} I(t)\sim kr\sqrt {{v^3}t(2r - vt)}. \end{equation*}

## Supplementary Material

nwz025_Supplemental_Files
