# Supplementary material for: SLIPS-TENG: robust triboelectric nanogenerator with optical and charge transparency using a slippery interface
Source: Natl Sci Rev. 2019 Mar 1;6(3):540–50. doi: 10.1093/nsr/nwz025 (PMC8291521; doi:10.1093/nsr/nwz025)
Supplement: nwz025_Supplemental_Files [file nwz025_supplemental_files.zip › NSR_SI.docx]

**Supplementary information**

**SLIPS-TENG: Robust triboelectric nanogenerator with optical and charge transparency using slippery interface**

Wanghuai Xu^1,2,#^, Xiaofeng Zhou^1,3,#^, Chonglei Hao^1,#^, Huanxi Zheng^1^, Yuan Liu^4^, Xiantong Yan^1^, Zhengbao Yang^1^, Michael Leung^5^, Xiao Cheng Zeng^4^, Ronald X. Xu^2^, Zuankai Wang^1,6,*^

^1^Department of Mechanical Engineering, City University of Hong Kong, Hong Kong 999077, China

^2^Department of Precision Machinery and Precision Instrumentation, University of Science and Technology of China, Hefei, Anhui 230026, China

^3^Shanghai Key Laboratory of Multidimensional Information Processing, Department of Electronic Engineering, East China Normal University, Shanghai 200241, China

^4^Department of Chemistry, University of Nebraska-Lincoln, Lincoln, NE 68588, US

^5^School of Energy and Environment, City University of Hong Kong, Hong Kong 999077, China

^6^Department of Materials Science and Engineering, City University of Hong Kong, Hong Kong 999077, China

#These authors contribute equally to this manuscript

*Corresponding author: zuanwang@cityu.edu.hk (Z.W.)

**SUPPLEMENTARY MOVIES**

**Supplementary Movie 1. Droplet dynamic behaviors on SLIPS-TENG and SHS-TENG.**

Droplet hitting on SLIPS-TENG always keeps intimate contact. In contrast, droplet hitting on SHS-TENG easily bounces off without the preferential contact with both electrodes. The distinct dynamics result in enhanced electrical output stability on SLIPS-TENG.

**Supplementary Movie 2. Molecular dynamics simulations.**

To clearly present the process of EDL formations at the atomic level, a trajectory of 600 ps with a step size 1ps is taken. Sodium ions are marked in yellow, layers with grey atoms and orange atoms represent PTFE and the thin lubricant layer, respectively.

**Supplementary Movie 3. Water energy harvest by SLIPS-TENG at 25 °C and -3 °C.**

Demonstration of lighting up of LED bulb arrays with the continuous flow of water droplets on SLIPS-TENG at ambient (25 °C) and low (-3 °C) temperatures, respectively.

**Supplementary Movie 4.** **Water energy harvest by SHS-TENG at 25 °C and -3 °C.**

At ambient condition (25 °C), the continuous flow of water droplets on SHS-TENG results in the lighting up of LED arrays. In contrast, there is no obvious lighting at -3 °C.

**Supplementary Movie 5. Operation of SLIPS-TENG under bending state**

Demonstration of SLIPS-TENG operating under bending state. The softness and flexibility of the SLIPS-TENG endows it to be adopted to various substrates with more complex configurations. The SLIPS-TENG can light up the LED bulb arrays with a measured output power ~200 nW.

**SUPPLEMENTARY FIGURES**

**Supplementary Figure 1.** SEM images of PTFE membrane and SLIPS. After infusing with the lubricant, the PTFE membrane with nanoscale porous fiber-like textures becomes smooth and liquid-like.

**Supplementary Figure 2.** Self-cleaning property of SLIPS-TENG. A contaminated surface with graphite powder of SLIPS-TENG can be cleaned by sliding droplets.

**Supplementary Figure 3.** Anti-wetting property of SLIPS-TENG to various liquids. (**a**) Optical images showing the pinning of both ethanol and vegetable oil droplets on SHS-TENG. (**b**) In contrast, both droplets can easily slide away from the surface of SLIPS-TENG, suggesting its excellent anti-wetting property.

**Supplementary Figure 4.** Characterization of relaxation time and current output under different frequency of incoming droplets. (**a**) The relaxation time for this V-t curve is about 8 s. (**b**) Time-resolved short-circuit current generated from 0.5 Hz and 1.5 Hz incoming droplets. In our measurement, the interval time of two incoming droplets are controlled to be shorter than the relaxation time of transferring charges for better data visualization in measurement equipment. By this way, each peak in the V-t and I-t curves is corresponding to individual specific incoming droplets without undesired interference. Otherwise, incoming droplets with high frequency will generate disordered V-t curves. However, the frequency of incoming droplet has no effect on the current output because of its near zero relaxation time. As shown in (**b**), the peak and shape of measured I-t curves for incoming droplets of 0.5 Hz and 1.5 Hz are identical. Note that in practical application, the frequency of incoming droplet has no effect on the real electricity generation.


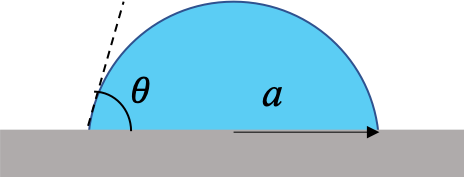


**Supplementary Figure 5.** Schematic of water droplet placed on a surface with contact angle *θ* and contact base radius *a*. For a water droplet with a given volume of *Ω* and a contact angle of *θ*, its contact base radius *a* and base contact area *S* can be expressed as

$a={[\frac{3\Omega\sin^{3}\theta}{\pi{(1-cos\theta)}^{2}(2+cos\theta)}]}^{\frac{1}{3}}$ and *S* ~ a^2^ ~ ${[\frac{\sin^{3}\theta}{{(1-cos\theta)}^{2}(2+cos\theta)}]}^{\frac{2}{3}}$ , respectively. In our experiments, the measured static contact angles of water droplet on SLIPS-TENG and SHS-TENG are around 109° ± 2.6° and 158° ± 4.4°, respectively. Thus, the ratio of contact area for SLIPS-TENG (*S*_SLIPS_) and SHS-TENG (*S*_SHS_) is calculated to be 7.7.

**Supplementary Figure 6.** Enhanced electricity generation stability in the electrical output at ambient temperature. **(a-b**) Schematic drawings showing the electricity generation on SLIPS-TENG and SHS-TENG, respectively. (**c-d**) Selected snapshots of droplet impacting on SLIPS-TENG and SHS-TENG, respectively. Clearly, droplet always keeps intimate contact with the SLIPS-TENG, whereas droplet hitting SHS-TENG easily bounces off without the preferential contact with underlying electrodes.

**Supplementary Figure 7.** Role of various interfaces on triboelectricity, which include the water/needle, water/ air, water/lubricant as well as lubricant/PTFE.

**Supplementary Figure 8.** Effect of SLIPS incarnation on charge generation. (**a**) Effects of membrane thickness and pore size of PTFE on charge generation. (**b**) The charge generation of droplet as a function of lubricant viscosity. (**c**) Comparison of generated charge for SLIPS-TENGs constructed by different lubricants (GPL103, silicone oil, and mineral oil). The thickness of over-coated lubricant layer (*h_0_*) on the PTFE membrane for all samples is maintained at ~ 2 µm.

**Supplementary Figure 9.** The time-dependent variation of lubricant layer thickness during the impact process. For the very thin lubricant layer, its thickness *h_0_* is almost unchanged. In all the cases, *h_0_* is much larger than the van der Waals length (dotted line in the figure).

**Supplementary Figure 10.** Charge distribution on PTFE and ITO by molecular dynamics (MD) simulation. (**a**) 100 negative charges with decent space are set on the first layer of PTFE (Top view). (**b**) 100 positive charge sites are set on the ITO electrode (Bottom view).

**Supplementary Figure 11.** Longevity of SLIPS-TENG. The average electric current and the lubricant mass loss as functions of the numbers of water droplets.

**Supplementary Figure 12.** Comparison of ice coverage ratio at low temperature (-3 °C). The inset shows that after 60 min of sequential dripping droplets, most SHS surface is covered with frozen droplets whereas the SLIPS-TENG surface is ice free.

**Supplementary Figure 13.** Electric output dependence on the load resistance. (**a)** Open-circuit voltages and short-circuit currents as a function of load resistance. (**b)** The output power as a function of load resistance.

**Supplementary Figure 14.** Rectified current output obtained from SLIPS-TENG under continuous water flow.

**Supplementary Figure 15.** Plot of time-dependent surface temperature after placing the SHS-TENG and SLIPS-TENG samples on a cooling plate with temperature fixed at -3 °C.

**Supplementary Figure 16.** Schematic drawing of the circuit diagrams for electricity generation analysis.
